# Electroconvulsive therapy after conservative treatment of vertebral fractures

**DOI:** 10.1192/j.eurpsy.2021.2067

**Published:** 2021-08-13

**Authors:** S. Martins, J. Quarenta, T. Teixeira, J. Pinheiro

**Affiliations:** 1 Departamento De Psiquiatria E Saúde Mental, Centro Hospitalar Tâmega e Sousa, Penafiel, Portugal; 2 Psychiatry Department, Centro Hospitalar entre o Tamega e Sousa EPE, Penafiel, Portugal

**Keywords:** ECT, Eletroconvulsive Therapy, Fractures

## Abstract

**Introduction:**

Electroconvulsive therapy (ECT) remains a valuable treatment for major depression with psychotic symptoms. However, it is necessary to pay special attention when there is a history of fractures.

**Objectives:**

Through the description of the following clinical case, we will emphasize the importance of screening for vertebral fractures within ECT and the different procedures that must be taken if that occurs.

**Methods:**

We undertook a narrative literature review by performing a search on PubMed for English-written articles. The query used was “Electroconvulsive Therapy” AND “Vertebral Fractures”.

**Results:**

A 71-year-old woman was admitted with an episode of psychotic depression. Basic tests were performed and were all normal. After not responding to pharmacologic treatment, she was referred for ECT. The patient had a full recovery after 4 weeks of biweekly sessions. She was discharged and proposed for maintenance ECT. However, she started complaining of back pain after falling and did an X-ray and CT scan which revealed fractured L1 and L2. It was suggested conservative treatment with a Jewett orthosis. Within this period, the ECT was suspended and after a 4-week treatment, the fracture was consolidated. As there was no risk of neurological compression, the treatment was restarted with the same dosage of succinylcholine, and it was achieved complete muscular relaxation. The patient fully recovered without any orthopedic sequel.

**Conclusions:**

Electroconvulsive therapy can be safely performed after conservative treatment of vertebral fractures, if special attention is provided to complete muscular relaxation. For this effect, the dosage of succinylcholine can be adjusted.

**Disclosure:**

No significant relationships.

